# 3D Laparoscopy-Assisted Operation to Adult Intussusceptions During Perioperative Period of Liver Transplantation: Case Report and Literature Review

**DOI:** 10.3389/fsurg.2021.647767

**Published:** 2021-06-21

**Authors:** Qing Gao, Shuo Yuan, Yang Li, Chao Chen, Xiaosun Liu, Jiren Yu

**Affiliations:** ^1^Department of Gastrointestinal Surgery, The First Affiliated Hospital, Zhejiang University School of Medicine, Hangzhou, China; ^2^Department of Gastrointestinal Surgery, Mingzhou Hospital, Zhejiang University, Ningbo, China

**Keywords:** adult intussusception, perioperative period, intestinal dysfunction, laparoscopy, case report

## Abstract

Adult intussusception during the perioperative period is defined as an extremely rare condition, especially when it emerges within short intervals of laparotomy, which may be explained as an unphysiological peristaltic function of the bowel without any lead points. Accurate diagnosis and therapeutic schedule predict a satisfactory outcome. Here, we introduce the case of a 32-year-old man who had symptoms of abdominal pain, no gas emission, and defecation shortly after liver transplantation. Intussusception was definitely diagnosed by abdominal CT, and then reduction was operated successfully by three-dimensional laparoscopy-assisted operation. There were no other complaints, and no secondary lesions were detected during the postoperative rehabilitation process in the hospital and over a follow-up period of 6 months. Relevant literature has been summarized subsequently. A promising minimally invasive surgery along with minimal secondary trauma was presented by laparoscopy in this rare case, providing the potential remedy for perioperative intussusception in the adult.

## Introduction

Adult intussusception is a rare entity and defined as the invagination of partial intestinal segment into an adjacent segment of the bowel (1). In a review study (2), it is estimated that ~2.5‰ of adult intussusception has been recorded in a medical center. There are two different categories, antegrade intussusception and retrograde intussusception, distinguished by the telescoping of either the proximal or distal portion into the opposite end of bowels. Unlike in pediatric patients, adult intussusception hardly presents with a triad of symptoms: acute abdominal pain, hematochezia, and palpable mass, and 70% of all cases are correlated with bowel obstruction, such as abdominal pain, distension, and constipation (3). Therefore, delayed diagnosis or even misdiagnosis resulting from non-specific symptoms remains high risk clinically. Most cases of intussusception in children are idiopathic, but almost all of the adult intussusception cases are closely linked to pathological intestines, which are tumor- or operation-related. However, in some cases, there are no apparent evidence that any intestinal lesions exist, partial adult intussusceptions are possibly associated with dysfunction of bowel motility among regional segments (1), which is commonly regarded as a more infrequent etiology in published reviews. The rare case of perioperative intussusception in adults has been mentioned below with symptoms of abdominal pain, constipation, and other signs, and 3D laparoscopic surgery was selected to repair the injury of the bowels after excluding any secondary factors. A relevant literature review will be discussed later.

## Case Presentation

**Figure 1:**
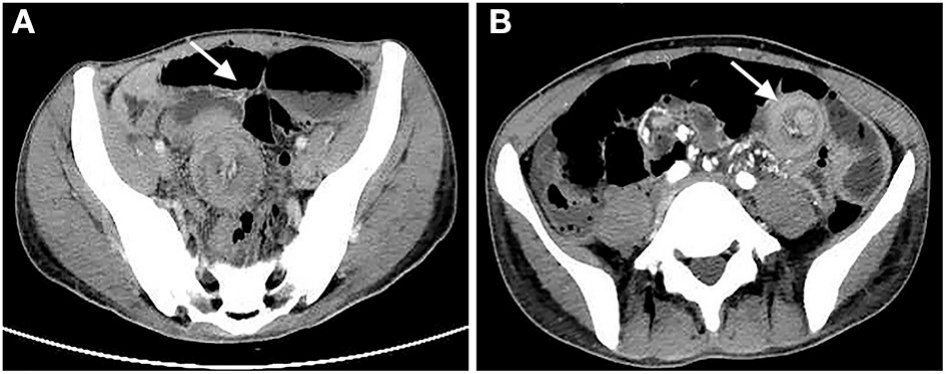
**(A)** Abdominal CT scan first noticed intestinal obstruction with sign of air-fluid level instead of intussusception. **(B)** Transverse section of CT scan indicated intussusception with sign of “target-like” or “concentric double ring” (both are indicated by white arrow above).

We present the case of a 32-year-old man who has just undergone liver transplantation for acute liver failure and decompensated liver cirrhosis. Chief complaints of slight abdominal distention and cramp pain in the left lower quadrant were referred during the postoperative period of 15 days. The patient had no other malaises of nausea, vomit, and bloody tool, only performed with descending frequency and shape alteration of defecation. Physical examination showed an apparent inverted T-shape surgical incision. Then, abdominal palpation of the left lower quadrant showed slight tenderness but there was rebound, muscle rigidity, or abdominal mass. An abdominal CT scan was completed few days later, which disclosed the sign of proximal enterostenosis instead of direct intestinal intussusceptions based on partial air-fluid level (Figure 1A). Conservative treatment, such as insertion of a gastric tube, NPO, and nutrition support was carried out for next the 4 days, but somatic complaints have not been visibly alleviated yet. The clinical symptoms were exacerbating progressively, and it was not until the patient failed to defecate and exhaust that the severity and urgency of these complaints were gradually recognized. CT scan was performed to evaluate for nosogenetic factors of intestinal obstruction again, which pointed out the apparent sign of “target-like” or “concentric double ring,” revealing the objectivity of intestinal intussusceptions (Figure 1B).

**Figure 2:**
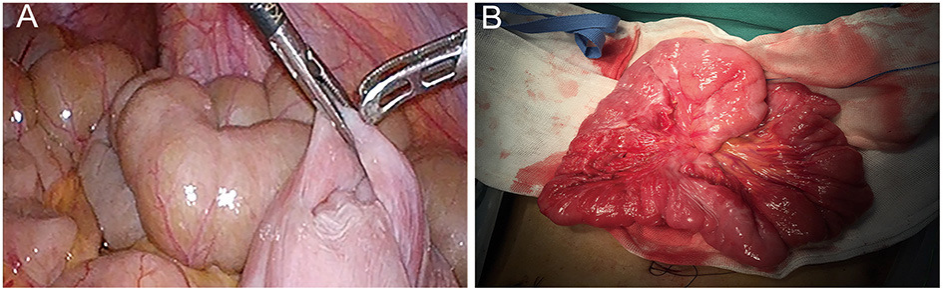
**(A)** Antegrade intussusception was observed directly through laparoscopic operation. **(B)** An intraoperative incision was made after laparoscopy in order to ascertain that adhesion among focal walls is restored, and that the layer of injured bowel serosa would be sutured integrally.

Then, surgical intervention was considered to diagnose precisely and solve the problem. Preoperative laboratory findings and vital signs revealed no specific abnormality. A 3D-laparoscopic exploratory operation and subsequent reduction were performed after excluding operative contraindication, which revealed antegrade intussusception that the proximal bowel invaginated into distal segment incidentally (Figure 2A). The lesion had a span of ~25 cm in the midst of normal bowels. Later, without large-scale postoperative adhesion or enteric abnormality found elsewhere, enterolysis and simple reduction *via* laparoscopy had been completed (Supplementary Video 1). In order to ascertain that local adhesion among focal walls was restored, and that the serosa layer could be repaired completely, a small intraoperative incision was made below the umbilicus, owing to the limitation in exercisable space and complicated stitching techniques under laparoscopy. No intestinal ischemia or necrosis was noticed after returning to physiological morphology (Figure 2B). Lasting for about 3 h, the whole operation was completed mostly by 3D laparoscopy.

**Figure 3:**
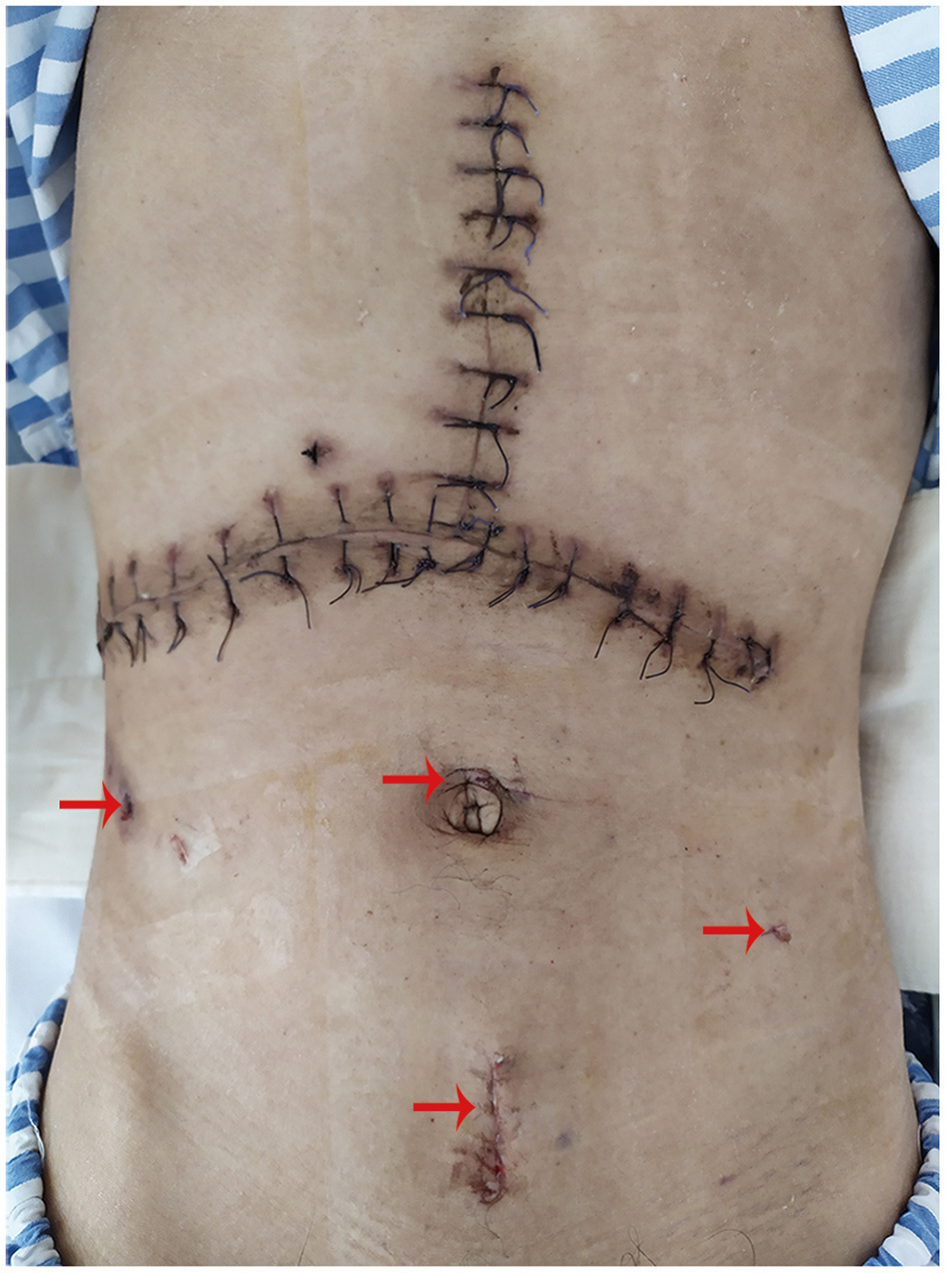
Small incisions (indicated by red arrow above) derived from laparoscopy and an inverted T-shape surgical incision caused by liver transplantation were revealed clearly.

The process described that the surgery featured with a small incision accompanied with mild trauma was different from conventional laparotomy (Figure 3). From slight complaints that evolved to a severe condition, until reduction was finished, the whole course of illness lasted more than 10 days. The symptoms of abdominal distension and pain were alleviated gradually. With expected postoperative recovery, the man was discharged 9 days later and no signs of obstruction were detected by abdominal CT scan (Supplementary Video 2). Additionally, there were no other complaints and positive signs during the follow-up every 2 months for 2 months in total.

## Discussion

Adult intussusception frequently presents with acute (<3 days), subacute (4–14 days), and chronic (>14 days) symptoms, most of which are involved in various features of digestive tract obstruction (4, 5). Intermittent abdominal pain is the most common cause of hospital admission among all complaints, as high as 77–90%, whereas others, such as jaundice, vomiting, obstipation, also become part of numerous symptoms (4, 6). Because of non-specific physical signs and disease duration, it is infeasible to diagnose intussusception in adults based on presentation and physical examination (3, 6). Early diagnosis during the course of the disease is still difficult, and merely a small number of cases could be detected before operation. Hence, a clinical decision is required to be made timely to ensure the vitality and physiological peristalsis of bowels, otherwise meeting with mesenteric ischemia for a long time and even enteric necrosis would be irreversible.

Main etiologies of pediatric and adult intussusception are attributed to be idiopathic or underlying intestinal lesions associated with anatomic or infectious factors, postoperative adhesion, and other benign or malignant neoplasms (1, 6, 7). A retrospective study shows that chronic symptoms over 14 days are perceived as an independent factor to predict malignancy, which demonstrates the importance of onset time of illness (4). There are no apparent lead points in about 90% of intussusception cases in children, and advancing age implicates miscellaneous pathogenic factors (6, 8). Conversely, malignant or benign tumors are still major points leading to adult intussusception, the morbidity of which is up to 50–70% reported in a meta-analysis and a review (3, 9). Also diverticulum, inflammatory bowel disease, and so on, occasionally have been mentioned in sporadic research studies (10, 11). Certainly, early post-operative intestinal occlusions could be relatively easy to identify, but the causes are multifactorial and ambiguous, which might be linked with primary surgical stress response, intraoperative iatrogenic mechanism, prolonged placement of abdominal drainages due to immunological response and inflammatory stimuli leading to edema of intestinal wall and exudation, electrolyte imbalance, and perioperative transfusion, etc. (12–14). Interestingly, much rarer etiologies of perioperative intussusception occurring shortly after liver transplantation in this case has not been reported in other reviews or cases, which may be explained as aberrant peristaltic activity and brief dysrhythmic contractions of intestines without any secondary factors (15, 16), being different from extensive postoperative adhesion of the bowels. Although adult intussusception has been described as an abnormality of bowel function, the exact pathogenesis of intussusception remains greatly unknown.

The diagnosis of intussusception in adults relies more on imaging techniques, because of untypical manifestations and latent structural lesions, which are usually relevant to the clinical outcomes of this emergency. Ultrasonography has unique advantages in diagnosing and screening the disease in children, treated as a non-radiation intervention with accuracy of 49.2–100% (9, 16), but physiological characteristics that massive air exists in the bowel loop and individual obesity, along with limitation in the experience of surgeons, have a negative impact on its quality and clinical practice in adults (17, 18). Also, an abdominal plain film has been advised to be an initial tool to diagnose intestinal obstruction characterized by the apparent air-fluid level, of which sensitivity is <50%, and fails to find out potential causes (8). Both of them play a role in the diagnosis of intestinal obstruction but also have own demerits. Abdominal CT has been actually considered to investigate the condition in similar cases. The characteristic signs of abdominal CT referred to intussusception in adults could be detected as “target-like” or “concentric double ring” on the transverse section or “sausage-shaped” on the coronal section based on a layer effect (1, 10, 17). The former has also been observed in the case. Furthermore, abdominal CT can reveal pathological bowels and their mesenteric vessels, along with the ability to locate luminal mass with detectable diameters and underlying points (17, 19). Although its diagnostic value is superior compared with ultrasonography and abdominal X-ray, especially in adults, it still cannot ignore the risk of missing or misdiagnosis as depicted by the first CT scan above, and a repeat disease evaluation would be of significance to guide explicit management. Besides, barium enema, ever regarded as the technique to diagnose and reduce intussusception, is gradually abandoned in adults, owing to complications, such as perforation, severe abdominal infection, and malignant cell dissemination (8, 19). From what has been discussed above, abdominal CT may be the valuable and normalized method for the diagnosis of adult intussusception.

Restoring the physiological properties and function of bowels has been deemed the ultimate intention, and there are distinct differences with regard to the treatment of pediatric and adult intussusception. Hydrostatic reduction, namely enema reduction, is still recommended for idiopathic intussusception in children with an 80–85% success rate (16). In contrast, for adult intussusception, the operation ought to be considered first not only because a majority of patients are treated by non-surgical reduction with high percentages of failure and recurrence (20), but the factors, such as irrigation pressure and duration, the existence of ischemic intestines, and a higher proportion of lesions, impose restrictions on its value. Obviously, the surgical reduction could work out with fairly well-recovery, supported by a systematic review that reduction shall be attempted superior to resection without evidence of suspicious malignant tumor regardless of hemodynamic disorder (9). Nevertheless, it is undeniable that either malignancy or benign neoplasm is mostly served as a lead point in adults, and non-excision may be exposure to the risk of implantation metastasis. In the majority of adult intussusception cases, resection is defined as the optimal method while the proportion of reduction merely occupy below 20% (2, 3, 7). With the advent of minimally invasive surgery, laparoscopy has been preferred increasingly by surgeons, depending on their expertise and the general conditions of a patient (17). It is reported that 71% of intussusception cases could be successfully restored by laparoscopy followed by a low rate of complications and shorter hospital stays compared with laparotomy, but it also presents some cases converted to laparotomy, on account of intestinal ischemia, poor visualization, and limited operative space (21, 22). Moreover, once postoperative ileus has been early perceived, it is indispensable that supportive care, such as NPO, nasogastric tube decompression, parenteral nutrition, and fluid resuscitation, should be considered first, and medications such as enterodynamic drugs, non-steroidal anti-inflammatory drugs (NSAIDs), glucocorticoid, and gut hormones have some value to ameliorate the symptoms without systematic evidence yet (14, 23, 24), but it still fails to recover gastrointestinal transit and switches to surgical intervention afterwards. Thus, although laparoscopic operation for intussusception in adults lacks strong evidence like randomized clinical trial, it is still worth more attempts to solve these emergencies, particularly during the perioperative period of first laparotomy with serious somatic and visceral stress. In addition, compared with 2D laparoscopy, 3D imaging possesses preferable depth perception and multidimensional anatomic level (25), which provide more creative methods and challenges for surgeons as well.

## Conclusions

In conclusion, adult intussusception during the perioperative period of liver transplantation is extremely rare, and it easily involves misdiagnosis and then gets worse results. Therefore, definite diagnosis through abdominal CT and relief of obstruction performed by laparoscopy exerts a positive influence on clinical outcomes. Although the patient has attained a good prognosis so far after laparoscopy-assisted reduction, a longer follow-up period and concrete evidence, such as imaging assessment, will be essential to demonstrate no recurrence and other secondary lesions.

## Data Availability Statement

The original contributions presented in the study are included in the article/Supplementary Material, further inquiries can be directed to the corresponding author/s.

## Ethics Statement

Written informed consent was obtained from the patient for publication of this case report.

## Author Contributions

QG wrote the original manuscript. SY offered some further advice for revision. XL mainly contributed to laparoscopic operation and introduction of the case. Collection of patient information and literature was performed by QG, CC, and YL. JY gave guidance in the submission of this study. All the authors read and approved the final manuscript.

## Conflict of Interest

The authors declare that the research was conducted in the absence of any commercial or financial relationships that could be construed as a potential conflict of interest.

## Supplementary Material

Supplementary Video 1 Enterolysis and simple reduction have been completed by the laparoscopic operation.

Supplementary Video 2 No signs of obstruction were detected by abdominal CT scan when patient was discharged.
